# Experience of virtual commissioning of a process control system for the production of high-paraffin oil

**DOI:** 10.1038/s41598-022-21778-0

**Published:** 2022-11-01

**Authors:** Yury V. Ilyushin, Vadim Fetisov

**Affiliations:** 1 Department of System Analysis and Management, Saint Petersburg Mining University, Saint Petersburg, Russia; 2 Department of Petroleum Engineering, Saint Petersburg Mining University, Saint Petersburg, Russia

**Keywords:** Energy science and technology, Mathematics and computing

## Abstract

This work describes the experience in developing and testing software for oil industry automation control systems based on the simulation of technological processes and control systems combined in virtual reality, this approach is called virtual commissioning and is widely used in the world both to create automated process control systems and to simulate interactions between different systems.

## Introduction

The twenty-first century can be described as the century of scientific and technological progress. The growth of industrial production requires a significant increase in the amount of raw ma-terials consumed.

The huge increase in the production of paraffinic and heavy crude oil due to the constant depletion of conventional oil reserves has attracted considerable attention from researchers. The production of waxy deposits in significant quantities can interfere with the flow ability of these waxy crude oils, which can eventually lead to production shut-downs^1,2^. The deposition and transformation of paraffin molecules into a paraffin gel, which occupies the cross-sectional area of the inner surface of the pipeline, occurs when the bulk oil temperature drops below the paraffin appearance temperature^3,4^. As a result of the accumulation of paraffin on the walls of the pipeline, the area of oil flow decreases. This phenomenon can lead to an emergency situation and a complete cessation of oil transportation through the pipeline^5^, which can lead to undesirable consequences of stopping production and large economic losses^6–8^. There are such consequences as the accumulation of paraffin deposits in a large amount, leading to the formation of a paraffin gel^9,10^, and resulting in aggregation of paraffin in the oil^11,12^ with changes in temperature and pressure; gelation of the paraffin layer, which mainly affects the poor flow ability of waxy crude oils^13,14^; layered deposition of wax, which leads to blockage of the flow line^15,16^ and which ultimately leads to a complete stop of production processes.

When paraffin is deposited in a pipeline, such mechanisms of molecular diffusion occur^17^ as the formation of paraffin crystal nuclei, Brownian motion, and diffusion shift^18^. When using a pour point depressant, such phenomena as eutectic adsorption occurs^19^. For example, in work^20^, the authors describe an experiment in which a polarizing microscope was used to observe the morphological lattice of paraffin crystals. In work^21^, the authors conducted an experiment in which wax crystals were formed and separated into three stages using a rheometer that was used when changes in the morphological state of oil occur, as a result of which a change in its viscosity occurs and it is necessary to carry out a physicochemical analysis.For this purpose such laboratory in-struments as a gas chromatograph and an infrared spectrometer and other technical methods for determining the quality and composition of oil are used, including both-physical and chemical methods^22–24^.

As an example, which is presented in most of the literature, this is a method for re-moving paraffin from the pipe walls using scrapers^25^;this performs the function of the mechanical friction of the scraper against the pipeline wall, as well as heating the pipe cavity^26,27^. When using pipeline heating, heating detectors and cables are installed on the oil heating pipeline, while the temperature of the heating cable or detector must be higher than the paraffin precipitation temperature^28,29^. Although it is widely used in oil fields^30–32^, the heating furnace has a large heat loss, which is not beneficial from either an economic^33^ or environmental point of view^34^. Physicochemical methods^35^ use the addition of some inhibitors to oil to prevent the formation of paraffin deposits^36,37^. These inhibitors and depressants improve the rheological properties of crude oil during pipeline transport^38–40^. Physico-chemical methods^35^ use the addition of certain inhibitors to oil to prevent the formation of paraffin deposits^36,37^; these inhibitors and pour point depressants^38–40^ such as comb polymers, polyvinyl acetate, amorphous polymers, surfactants and nanohybrids, improve the rheological properties of crude oil in pipeline transport^41–43^. These paraffins are a mixture of alkanes^44–46^, which consist of straight and branched chains^47,48^, solid or liquid state^49,50^. These include microcrystalline paraffin deposits^51^ with a carbon chain from C_16_ to C_40_ (alkenyl radicals), as well as isoparaffin hydrocarbons, the content of which is from C_30_ to C_60_^52,53^ and which crystallizes into the amorphous structure of the naphthenic ring.

The main tasks aimed at improving the economic efficiency of oil production are the study of linear automatic oil production systems, the construction of their optimal structures, the search for methods and control algorithms^54^, and the patterns of optimal functioning of the technological process.

The purpose of this work is to develop a control system for the process of pulsed heating of a high-paraffin oil flow in the tubing of low-rate oil wells, aimed at reducing the cost of oil production by preventing the formation of asphalt, tar, and paraffin deposits.

## Methodology

### Existing solution method

**Figure 1:**
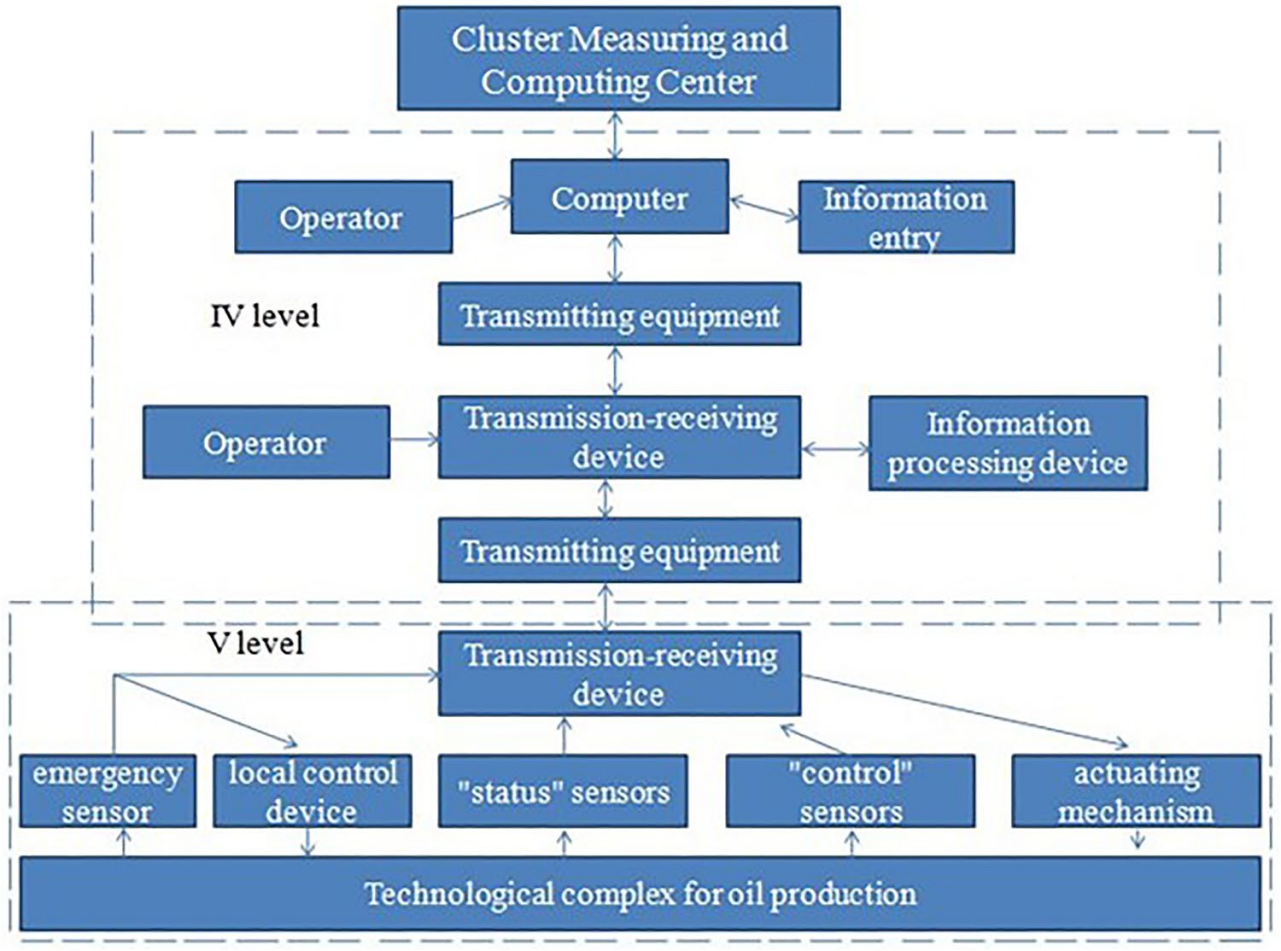
Scheme of information flows in the oil and gas production department in terms of technological complexes for oil production.

Analyzing the oil field as an object of management, it is possible to identify a five-level structure of information interaction in the oil field, which has three adminis-trative levels and two operational levels^55^. The cluster measuring and computing center, which is at the head of the operational level, supplies the central engineering ser-vice in real time with operational information about the technical condition and modes of operation of the exploited field, Fig. 1. In the process of applying actions to remove Asphalt-resinous and paraffin deposits in this information scheme, nothing changes, since the technological process for removing Asphalt-resinous and paraffin deposits is not part of the general technological process, but is a third-party, one-time procedure. We will carry out a deep modernization of the technological process by integrating means preventing the formation of Asphalt-resinous and paraffin deposits into the technological process.

**Figure 2:**
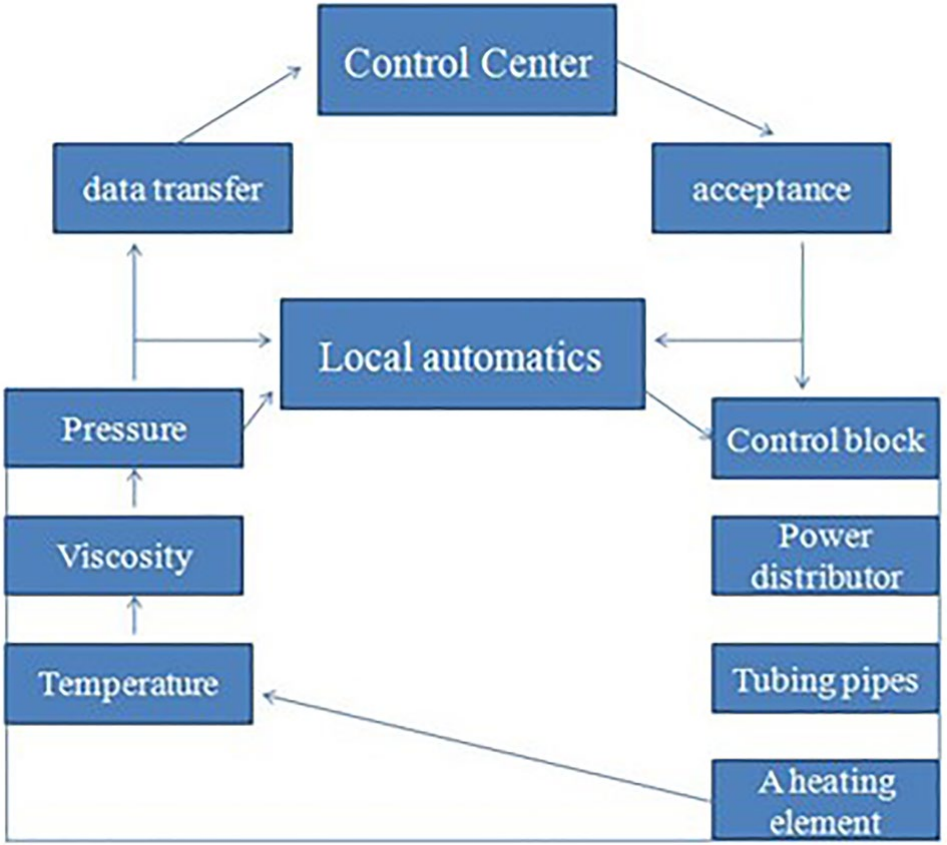
Scheme of information flows of an oil well with installed heating elements.

Thus, the fifth level of the conceptual model will take the form shown in Fig. 2.

The solution to the key element of this upgrade is the introduction of a pulsed heating element into the system. With this element, the classical mathematical model of the tubing has the form:

1 $$\begin{gathered} \frac{\partial T}{{\partial t}} = a^{2} \left( {\frac{{\partial^{2} T}}{{\partial x^{2} }} + \frac{{\partial^{2} T}}{{\partial y^{2} }} + \frac{{\partial^{2} T}}{{\partial z^{2} }}} \right); \hfill \\ 0 < x < l_{x} ; \, 0 < y < l_{y} ; \, 0 < z < l_{z}. \hfill \\ \end{gathered}$$

2 $$\begin{gathered} T(x,y,L_{z} \tau ) = U(x,y,\tau ); \, \frac{\partial T(x,y,0,\tau )}{{\partial z}} = 0; \hfill \\ T(x,0,z,\tau ) = T(x,l_{y} ,z,\tau ) = T(0,y,z,\tau ) = T(l_{x} ,y,z,\tau ) = 0; \hfill \\ T(x,y,z,0) = 0. \hfill \\ \end{gathered}$$

The equation can be represented as a Green’s function.

### Mathematical model development

We will obtain dynamic mathematical models to describe the temperature field of oil flow in tubing.

A one-dimensional equation is presented:

3 $$\begin{aligned} T(x_{j} ,t) & = \sum\limits_{i = 1}^{d} {} \sum\limits_{n = 1}^{k} {\frac{2}{l}\exp \left[ { - \left( {\frac{\pi{na}}{l}} \right)^{2} t} \right]} \sin \frac{\pi{n}}{l}x_{j} \sin \frac{\pi{n}}{l}\xi_{i} \\ & \quad + \sum\limits_{p} {} \sum\limits_{n = 1}^{k} \frac{2}{l} \exp \left[ { - \left( {\frac{\pi{na}}{l}} \right)^{2} \left( {t - \tau_{p} } \right)} \right]\sin \frac{\pi{n}}{l}x_{j} \sin \frac{\pi{n}}{l}\xi_{z\left( p \right)}. \\ \end{aligned}$$

Two-dimensional equation:

4 $$\begin{aligned} T(x_{j} ,y_{j} ,t) & = \sum\limits_{i = 1}^{d} {} \sum\limits_{k,m = 1}^{\infty } {\frac{4}{{l_{1} \cdot l_{2} }}\exp \left[ { - a^{2} \pi^{2} \cdot t \cdot \left( {\frac{{k^{2} }}{{l_{1}^{2} }} + \frac{{m^{2} }}{{l_{2}^{2} }}} \right)} \right] \cdot \sin \left( {\frac{{k \cdot \pi \cdot x_{j} }}{{l_{1} }}} \right)} \cdot \sin \left( {\frac{{k \cdot \pi \cdot \rho_{i} }}{{l_{1} }}} \right) \\ & \quad \times \sin \left( {\frac{{m \cdot \pi \cdot y_{j} }}{{l_{2} }}} \right) \cdot \sin \left( {\frac{{m \cdot \pi \cdot \nu_{i} }}{{l_{2} }}} \right) + \sum\limits_{p} {\sum\limits_{k,m = 1}^{\infty } {\frac{4}{{l_{1} \cdot l_{2} }} \cdot } } \exp \left[ { - a^{2} \pi^{2} \cdot (t - \tau_{p} ) \cdot \left( {\frac{{k^{2} }}{{l_{1}^{2} }} + \frac{{m^{2} }}{{l_{2}^{2} }}} \right)} \right] \\ & \quad \times \sin \left( {\frac{{m \cdot \pi \cdot y_{j} }}{{l_{2} }}} \right) \cdot \sin \left( {\frac{{k \cdot \pi \cdot x_{j} }}{{l_{1} }}} \right) \cdot \sin \left( {\frac{{k \cdot \pi \cdot \rho_{z(p)} }}{{l_{1} }}} \right) \cdot \sin \left( {\frac{{m \cdot \pi \cdot \nu_{z(p)} }}{{l_{2} }}} \right). \\ \end{aligned}$$

Equation of a Line in Three Dimensions

5 $$G(x,y,z,\rho ,\nu ,\vartheta ,t) = \frac{8}{{l_{1} \cdot l_{2} \cdot l_{3} }} \cdot \sum\limits_{k,m,n = 1}^{\infty } {B_{k,m,n} } ( \cdot ) \cdot \exp \left[ { - a^{2} \pi^{2} \cdot t \cdot \left( {\frac{{k^{2} }}{{l_{1}^{2} }} + \frac{{m^{2} }}{{l_{2}^{2} }} + \frac{{n^{2} }}{{l_{3}^{2} }}} \right)} \right]$$

6 $$B_{k,m,n} ( \cdot ) = \sin \left( {\frac{k \cdot \pi \cdot x}{{l_{1} }}} \right) \cdot \sin \left( {\frac{m \cdot \pi \cdot y}{{l_{2} }}} \right) \cdot \sin \left( {\frac{m \cdot \pi \cdot z}{{l_{3} }}} \right) \cdot \sin \left( {\frac{k \cdot \pi \cdot \rho }{{l_{1} }}} \right) \cdot \sin \left( {\frac{k \cdot \pi \cdot v}{{l_{2} }}} \right) \cdot \sin \left( {\frac{n \cdot \pi \cdot \vartheta }{{l_{3} }}} \right).$$

**Figure 3:**
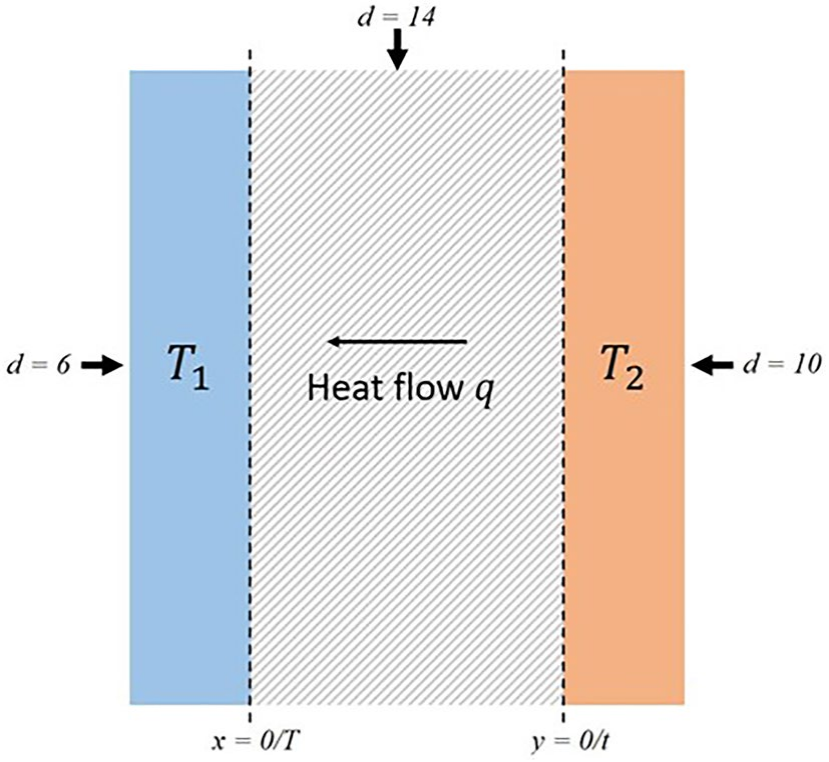
Temperature field values depending on time.

The dependences obtained allow us to carry out numerical simulation of the behavior of the temperature field in time^55,56^. For a one-dimensional equation, the graph of the dynamic change in the temperature field depending on the number of heating elements should look like the one shown in Fig. 3.

At the Fig. 3 shows the formed temperature field T_1_ and T_2_, which is significantly higher than the specified temperature regime. And the greater the number of heating elements d, the higher the temperature field.

### Simulation method

**Figure 4:**
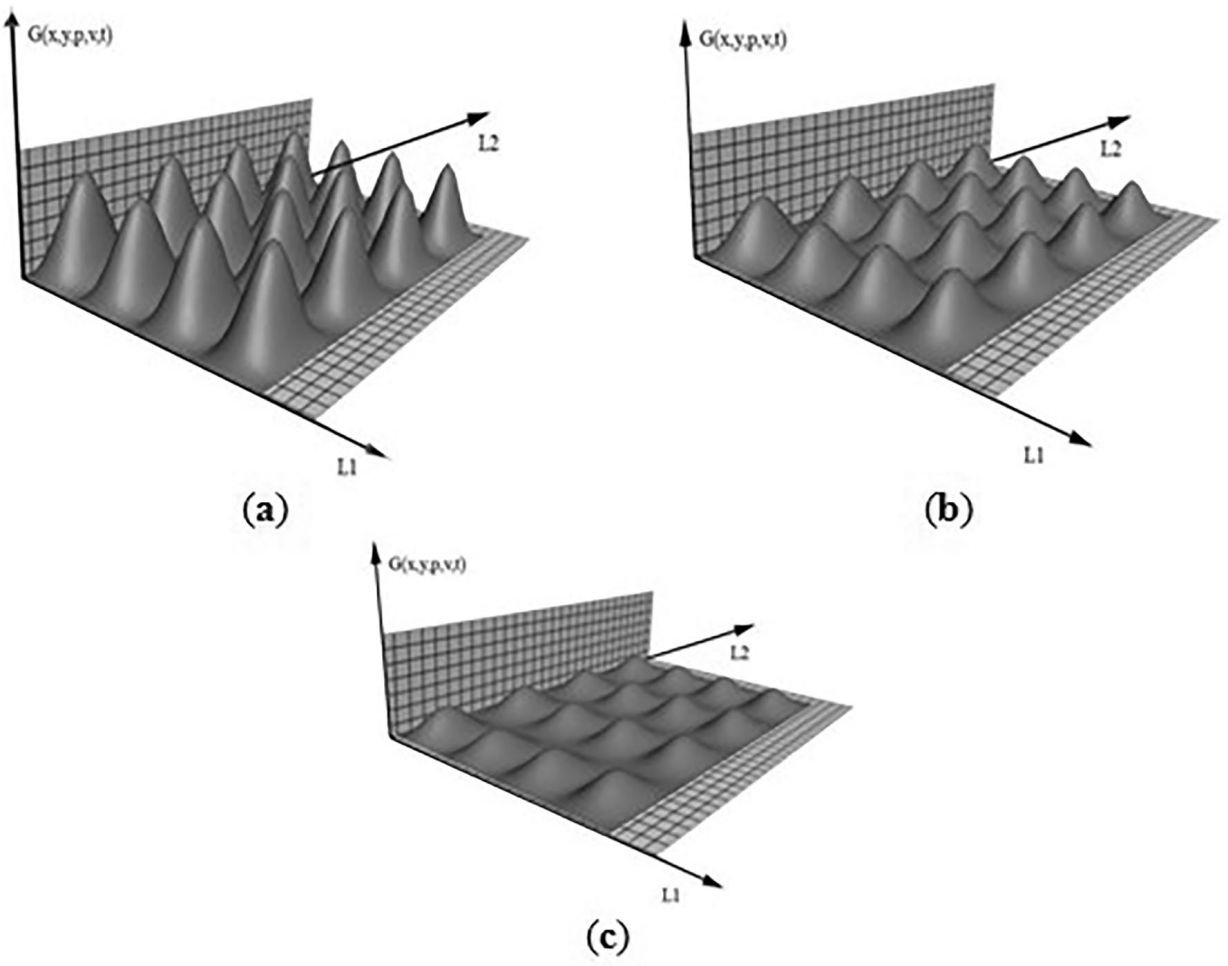
Dynamic change of the temperature field depending on time. (**a**) before change; (**b**) cycle change; (**c**) after change (RAD Studio 11.1 programming environment Delphi https://www.embarcadero.com/products/rad-studio).

In this work, programming in the DELPHI environment was used for a two-dimensional object for controlling the temperature field of oil flow between paraffin molecules in tubing. The graph of the function is shown in Fig. 4a–c.

Figure 4 shows that the generated temperature field has a cyclic nature, and the obtained control algorithms do not use all the heating elements located on the control object^56^. This Figure was made according to the developed mathematical model based on the Green’s function which allows you to create a point temperature effect of unit power. The existing Green’s function is one-dimensional. The author obtained a two-dimensional model of the optimal oil production regime.

Thus, it is possible to determine the optimal (smallest) number of heating elements required to maintain a given temperature regime^57^.

## Results

### Numerical example

For this, a mathematical model was obtained to determine the places and moments of switching on the heating elements based on the formulated optimality criterion for the two-dimensional case.

7 $$x = \arcsin \frac{{\frac{4}{{l_{1} l_{2} }}\sum\limits_{k,m = 1}^{\infty } {\sin \left( {\frac{m\pi{y}}{{l_{2} }}} \right)\sin \left( {\frac{k\pi \rho }{{l_{1} }}} \right)\sin \left( {\frac{m\pi{\nu} }{{l_{2} }}} \right)\exp \left[ { - a^{2} \pi^{2} t\left( {\frac{{k^{2} }}{{l_{1}^{2} }} + \frac{{m^{2} }}{{l_{2}^{2} }}} \right)} \right]} }}{{G\left( {x,y,\rho ,\nu ,t} \right)}}\left( {\frac{{l_{1} }}{k\pi }} \right);$$

8 $$y = \arcsin \frac{{\frac{4}{{l_{1} l_{2} }}\sum\limits_{k,m = 1}^{\infty } {\sin \left( {\frac{m\pi{x}}{{l_{1} }}} \right)\sin \left( {\frac{k\pi \rho }{{l_{1} }}} \right)\sin \left( {\frac{m\pi{\nu} }{{l_{2} }}} \right)\exp \left[ { - a^{2} \pi^{2} t\left( {\frac{{k^{2} }}{{l_{1}^{2} }} + \frac{{m^{2} }}{{l_{2}^{2} }}} \right)} \right]} }}{{G\left( {x,y,\rho ,\nu ,t} \right)}}\left( {\frac{{l_{2} }}{m\pi }} \right).$$

9 $$t = \frac{{\ln \left( {\frac{{\frac{4}{{l_{1} l_{2} }}\sum\limits_{k,m = 1}^{\infty } {\sin \left( {\frac{k\pi{x}}{{l_{1} }}} \right)\sin \left( {\frac{m\pi{y}}{{l_{2} }}} \right)\sin \left( {\frac{k\pi \rho }{{l_{1} }}} \right)\sin \left( {\frac{m\pi{\nu} }{{l_{2} }}} \right)\exp \left[ { - a^{2} \pi^{2} t\left( {\frac{{k^{2} }}{{l_{1}^{2} }} + \frac{{m^{2} }}{{l_{2}^{2} }}} \right)} \right]} }}{{G\left( {x,y,\rho ,\nu ,t} \right)}}} \right)\left( {\frac{{l_{1}^{2} }}{{k^{2} }} + \frac{{l_{2}^{2} }}{{m^{2} }}} \right)}}{{a^{2} \pi^{2} }}.$$

And three-dimensional case

10 $$\begin{aligned} x & = \frac{{l_{1} }}{\pi }\arcsin \frac{{\frac{8}{{l_{1} l_{2} l_{3} }}\exp \left[ { - a^{2} \pi^{2} t\left( {\frac{1}{{l_{1}^{2} }} + \frac{1}{{l_{2}^{2} }} + \frac{1}{{l_{3}^{2} }}} \right)} \right] \cdot \sin \frac{\pi }{{l_{2} }}y \cdot \sin \frac{\pi }{{l_{3} }}z \cdot \sum\limits_{i = 1}^{d} {\sin \frac{\pi }{{l_{1} }}} \rho_{i} \cdot \sin \frac{\pi }{{l_{2} }}\nu_{i} \cdot \sin \frac{\pi }{{l_{3} }}\vartheta_{i} }}{T(x,y,z,t)} \cdot \\ y & = \frac{{l_{2} }}{\pi } \cdot \arcsin \frac{{\frac{8}{{l_{1} l_{2} l_{3} }}\exp \left[ { - a^{2} \pi^{2} t\left( {\frac{1}{{l_{1}^{2} }} + \frac{1}{{l_{2}^{2} }} + \frac{1}{{l_{3}^{2} }}} \right)} \right] \cdot \sin \frac{\pi }{{l_{1} }}x \cdot \sin \frac{\pi }{{l_{3} }}z \cdot \sum\limits_{i = 1}^{d} {\sin \frac{\pi }{{l_{1} }}} \rho_{i} \cdot \sin \frac{\pi }{{l_{2} }}\nu_{i} \cdot \sin \frac{\pi }{{l_{3} }}\vartheta_{i} }}{T(x,y,z,t)}. \\ z & = \frac{{l_{3} }}{\pi }.\arcsin \frac{{\frac{8}{{l_{1} l_{2} l_{3} }}\exp \left[ { - a^{2} \pi^{2} t\left( {\frac{1}{{l_{1}^{2} }} + \frac{1}{{l_{2}^{2} }} + \frac{1}{{l_{3}^{2} }}} \right)} \right] \cdot \sin \frac{\pi }{{l_{1} }}x \cdot \sin \frac{\pi }{{l_{2} }}y \cdot \sum\limits_{i = 1}^{d} {\sin \frac{\pi }{{l_{1} }}} \rho_{i} \cdot \sin \frac{\pi }{{l_{2} }}\nu_{i} \cdot \sin \frac{\pi }{{l_{3} }}\vartheta_{i} }}{T(x,y,z,t)} \cdot \\ \end{aligned}$$

On the basis of these equations, a large number of computer and natural experiments were carried out, which showed the occurrence of thermal deformation of the tubing. To analyze thermal deformation, we will analyze the mathematical model.

11 $$\frac{{dT_{1} (x,r,\Theta ,\tau )}}{d\tau } = a_{1} \cdot \left( {\frac{{d^{2} T_{1} (x,r,\Theta ,\tau )}}{{dr^{2} }} + \frac{1}{r} \cdot \frac{{dT_{1} (x,r,\Theta ,\tau )}}{dr} + \frac{{d^{2} T_{1} (x,r,\Theta ,\tau )}}{{dx^{2} }} + \frac{1}{r} \cdot \frac{{d^{2} T_{1} (x,r,\Theta ,\tau )}}{{d\Theta^{2} }}} \right),$$

12 $$0 < {\text{x}} < {\text{ L}},{\text{ R2}} < {\text{r}} < {\text{ R1}}, \, 0 < \Theta < {36}0^{ \circ }.$$

Temperature field of oil flow

13 $$\frac{{dT_{2} (x,r,\Theta ,\tau )}}{d\tau } = a_{2} \cdot \left( {\frac{{d^{2} T_{2} (x,r,\Theta ,\tau )}}{{dr^{2} }} + \frac{1}{r} \cdot \frac{{dT_{2} (x,r,\Theta ,\tau )}}{dr} + \frac{{d^{2} T_{2} (x,r,\Theta ,\tau )}}{{dx^{2} }} + \frac{1}{r} \cdot \frac{{d^{2} T_{2} (x,r,\Theta ,\tau )}}{{d\Theta^{2} }}} \right),$$

14 $$0 < {\text{x}} < {\text{ L}}, \, 0 < {\text{r}} < {\text{ R2}}, \, 0 < \Theta < {36}0^{ \circ }.$$

Boundary conditions for the phase variable *T*_1_

15 $$\lambda_{1} \frac{{dT_{1} (x,R_{1} ,\Theta ,\tau )}}{dr} = \lambda_{B} \frac{{dT_{B} (x,R_{1} ,\Theta ,\tau )}}{dr},$$

16 $$T_{1} (x,R_{1} ,\Theta ,\tau ) = T_{B} (x,R_{1} ,\Theta ,\tau );0 < x < L, \, 0 < \Theta < 360^{ \circ }.$$

Boundary conditions for the phase variable *T*_2_

17 $$\lambda_{1} \frac{{dT_{1} (x,R_{2} ,\Theta ,\tau )}}{dr} = \lambda_{2} \frac{{dT_{2} (x,R_{2} ,\Theta ,\tau )}}{dr};$$

18 $$T_{1} (x,R_{2} ,\Theta ,\tau ) = T_{2} (x,R_{2} ,\Theta ,\tau );$$

19 $$0 < {\text{x}} < {\text{ L}}, \, 0 < \Theta < {36}0^{ \circ }$$

End faces of the object

20 $$\begin{gathered} T_{1} (0,r,\Theta ,\tau ) = T_{1} (L,r,\Theta ,\tau ) = 0;R{}_{2} < r < R_{1} ; \hfill \\ T_{2} (0,r,\Theta ,\tau ) = T_{2} (L,r,\Theta ,\tau ) = 0;0 < r < R_{2} ; \hfill \\ \end{gathered}$$

Then, the final equation for estimating the thermal deformation of the tubing wellbore has the form

21 $${\text{f}} = \sum\limits_{{{\text{j}} = {1}}}^{N} {{\text{arctg((}}\Delta {\text{x}}_{{\text{j}}} { - }\Delta {\text{x)/(2R}}_{{3}} {))}}.$$

### Thermodynamic properties

The final step in the assessment of the regulatory system is the determination of sustainability. It should be noted that the control system is non-linear, and therefore there are no methods for assessing stability. We adapt the Nyquist stability criterion for an impulsive distributed system. To do this, we obtain the transfer function of an open-loop control system, which is written as follows.

22 $$\begin{aligned} W^{*}_{P} \left( {G_{\eta,\gamma },s} \right) & = (1 - \exp ( - st) \, \cdot \frac{1}{s} \cdot \frac{1}{t} \cdot \sum\limits_{r = - \infty }^{r = \infty } {\left( {\left( {E_{1} \cdot \left[ {\frac{{n_{1} - 1}}{{n_{1} }} + \frac{1}{{n_{1} }} \cdot G_{\eta,\gamma } } \right] + E_{4} \cdot \left[ {\frac{{n_{4} - 1}}{{n_{4} }} + \frac{1}{{n_{4} }} \cdot G_{\eta,\gamma } } \right] \cdot \frac{1}{s} + E_{2} \cdot \left[ {\frac{{n_{2} - 1}}{{n_{2} }} + \frac{1}{{n_{2} }} \cdot G_{\eta,\gamma } } \right] \cdot s} \right) \cdot \frac{{\exp \left( {\beta (G_{\eta,\gamma } \cdot z^{*} } \right) + \exp \left( { - \beta (G_{\eta,\gamma } \cdot z^{*} } \right)}}{{\lambda \cdot \beta (G_{\eta,\gamma } ) \cdot \left( {\exp \left( {\beta (G_{\eta,\gamma } ) \cdot z_{L} } \right) - \exp \left( { - \beta (G_{\eta,\gamma } ) \cdot z_{L} } \right)} \right)}}} \right)}, \\ \beta & = \left( {\frac{{s + jr\omega_{u} }}{a} + G_{\eta,\gamma } } \right)^{1/2},\left( {\eta,\gamma = \overline{1,\infty } } \right) \\ \end{aligned}$$

**Figure 5:**
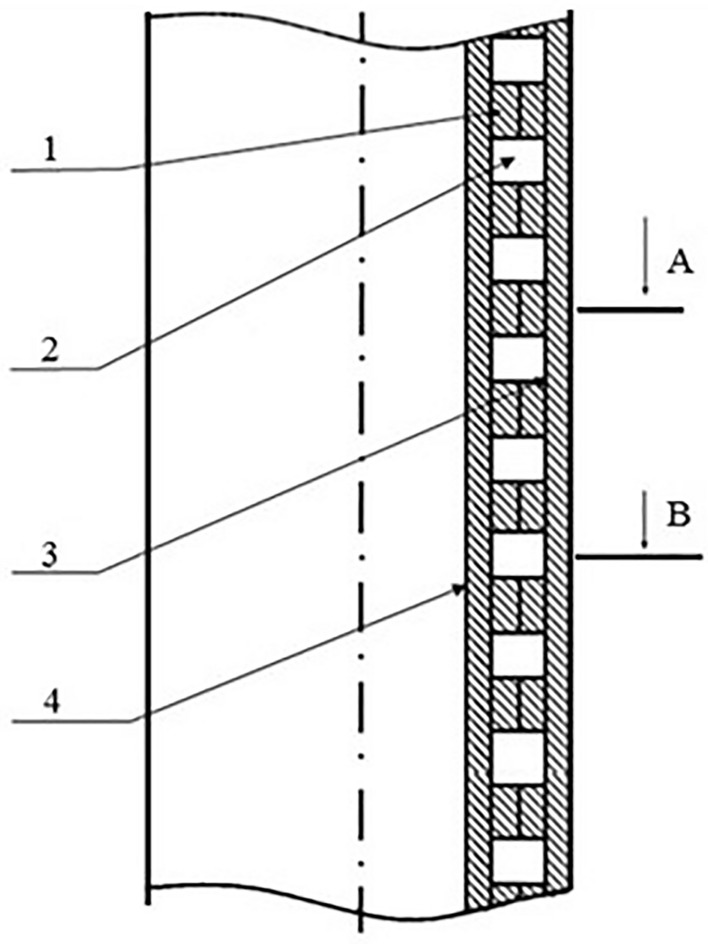
Scheme of tubing with impulse sectional heaters.

By passing from an infinite number of circles of unit radius, we obtain the hodograph of a spatially distributed impulse control system, Fig. 5.

Sectional heating elements are controlled by a programmable unit, Fig. 5, consisting of four arithmetic logic units.

**Figure 6:**
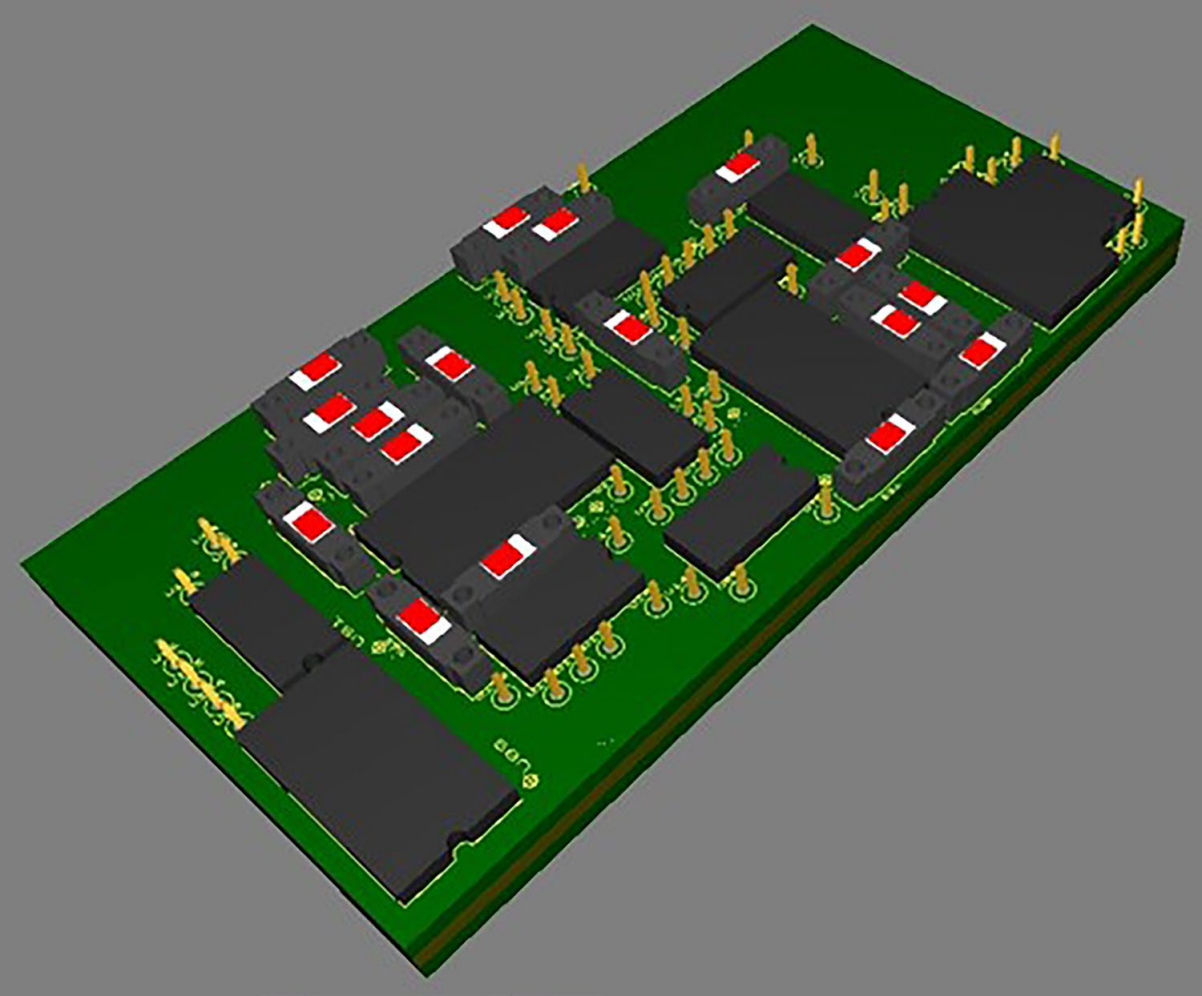
Layout model of parallel computing unit (top view). (Multisim https://www.ni.com/shop/electronic-test-instrumentation/application-software-for-electronic-test-and-instrumentation-category/what-is-multisim.html).

These logical elements are connected in parallel, which ensures a high speed of generating command signals. Figure 6 shows the main control circuit with control signal output indicators.

In addition, on the basis of the methods obtained, a number of thermal drills were obtained and tested on an industrial scale, performing various specific tasks of exploration and thermal drilling in the conditions of the Arctic zone. Experimental studies were carried out on an electron microscope in various modes, demonstrating uniform heating of the metal. The results of the experiment confirm the absence of destruction of the metal structure caused by pulsed heating.

## Discussions

As part of the study, a tubing with impulse sectional heaters was developed, consisting of external and internal casings and impulse-type heating elements installed between them, separated by bulkheads with a current-carrying channel inside. The principle of operation of this device can be divided into two modes: static and dynamic.

In static mode, the tubing string with impulse sectional heaters is not connected to the power supply. In this mode, the electric current is not supplied to the heaters, the temperature field is not formed. All structural elements located on a metal sleeve are at rest at ambient temperature. Oil rising through such a tubing cools, its rheological properties change, it becomes more viscous. Asphalt, resin and paraffin deposits begin to form on the tubing walls (Supplementary 1 Video). Asphalt-resin-paraffin deposits increase over time, which leads to a complete overlap of the column.

In dynamic mode, the tubing string is connected to the power supply network. In this mode, a pulsed current is applied to the heating elements and the temperature rises. Over time, the heating of the metal sleeve, the bulkhead and the entire structure as a whole begins. The tubing is heated above the paraffin temperature. Thus, asphalt-resin-paraffin deposits do not form on the tubing walls. Oil continues to rise along the tubing.

When compared with constant heating, which forms a temperature field, energy costs are reduced up to 4…6 times. It is important to note that the number and extent of installation of such heaters is not limited (Supplementary 2).

The methods presented above made it possible to obtain the optimal number of heating elements based on a given temperature regime. The production process at each field is unique and requires a specific approach to it, so there is no need to create a tubing block for each field individually. Thus, it is expedient to create a unified tubing that provides any technological regime. In this regard, in the presented technical device, the heating elements are located equidistantly at the minimum possible distance, without prejudice to the design features. The developed tubing is suitable for the extraction of highly paraffinic oil from any field.

**Figure 7:**
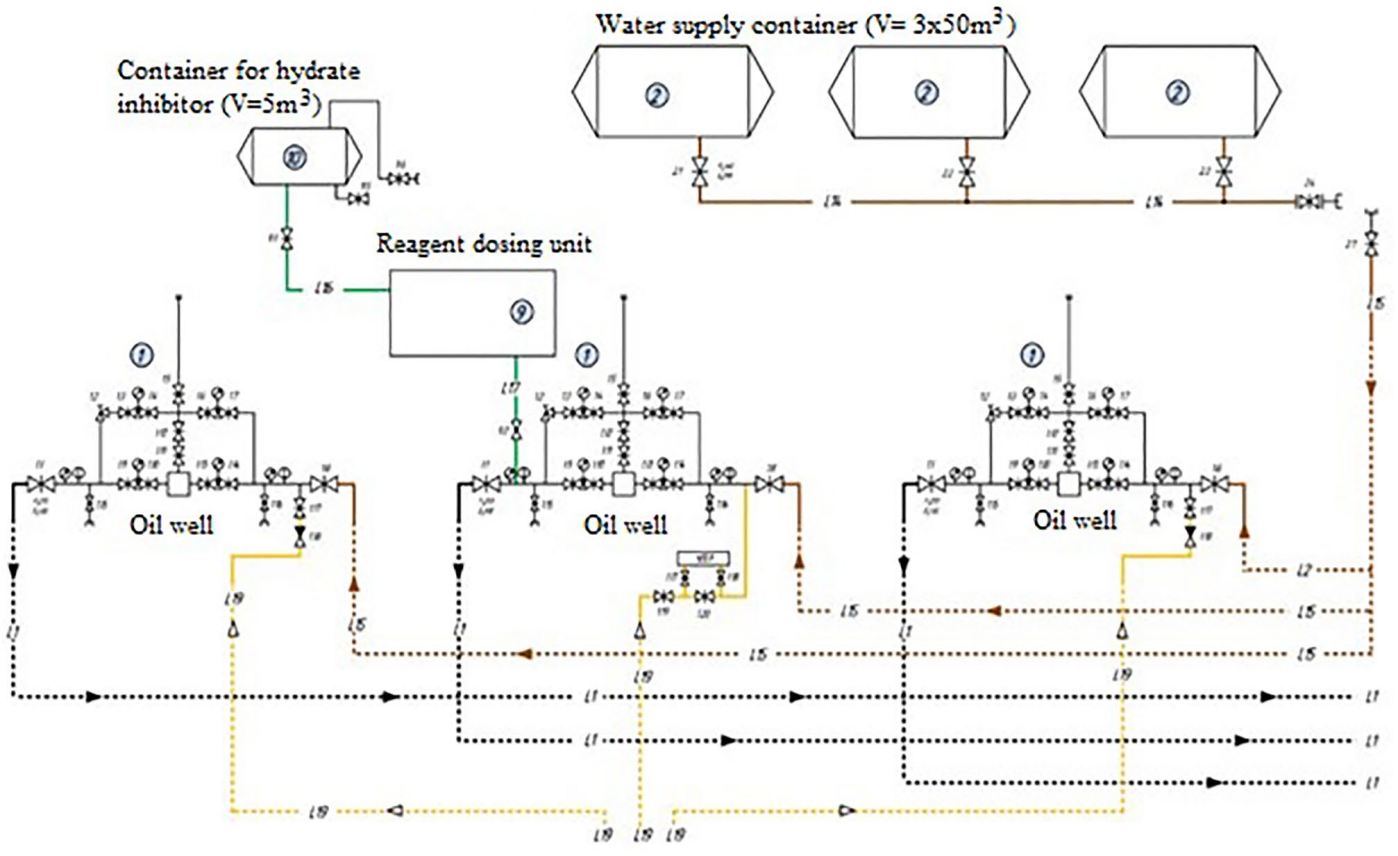
The existing scheme of automation of the technological process.

To prevent paraffin formation and clogging of tubing in the field under consideration, installations for supplying a hydrate formation inhibitor are used, Fig. 7. The installation includes:

- consumable capacity (V = 5 m^3^);
- consumable capacity (Apparatus 1–50-2400, V = 50 m^3^);
- technological block for inhibitor supply (pumping blocks);
- dosing pumps.

The hydrate formation inhibitor is supplied to the wells using dosing pumps from pre-filled containers V = 5m^3^ or V = 50m^3^.

**Figure 8:**
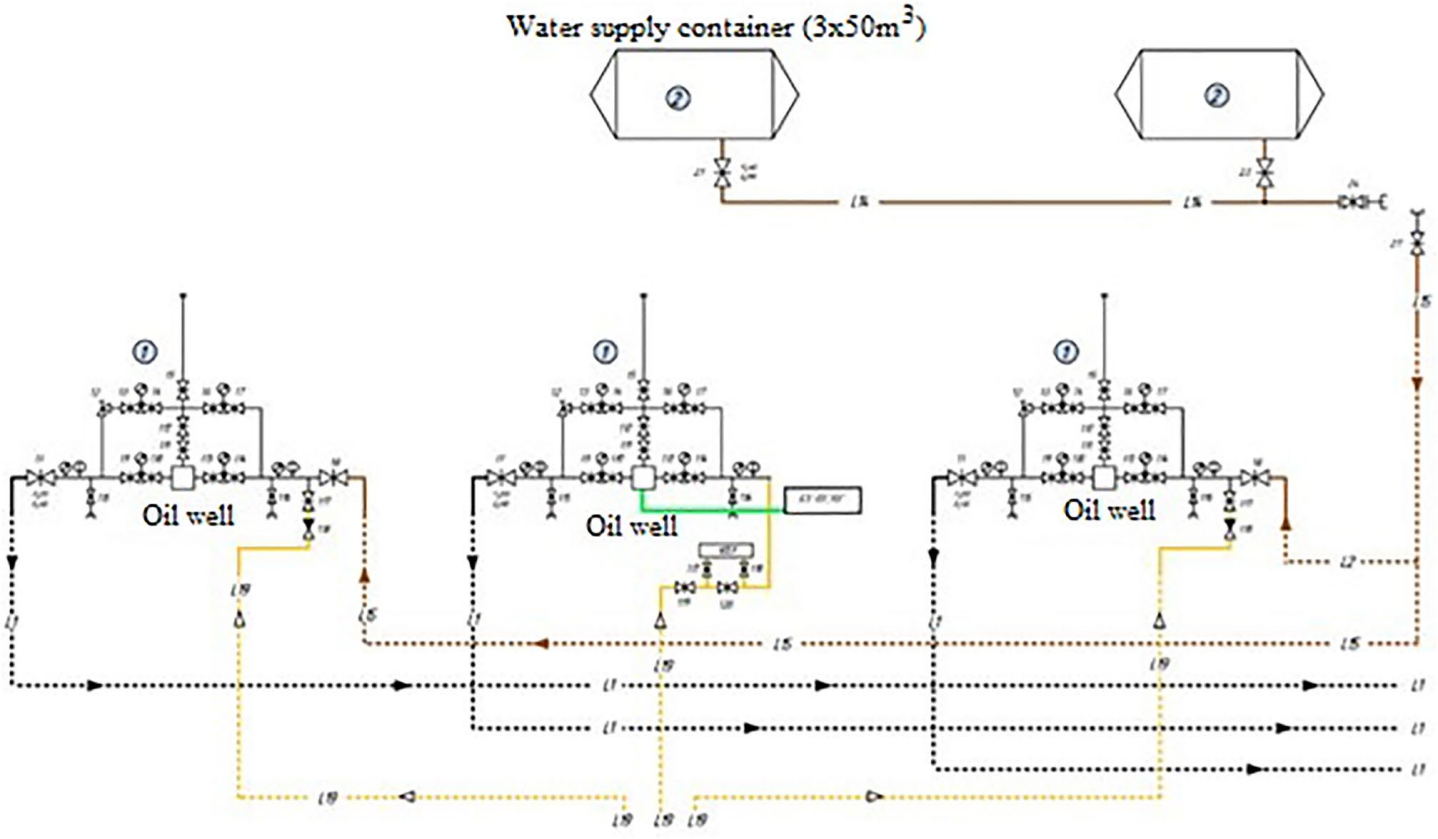
Proposed automation scheme.

After the implementation of the developed automation scheme, the technological process will be carried out as follows. All elements that ensure the injection of the inhibitor, both into the tubing itself and into the main pipeline, are removed from the automation scheme. The extracted oil will have a higher temperature, which will ensure that it does not need to be further diluted. This will have the economic effect of saving money on inhibitor storage tank rental and logistics costs. The elements of heating the heating cable are removed, in view of its exclusion from the circuit. At the same time, the existing automation scheme is being retrofitted with a controller for controlling pulsed sectional heaters, and power supply loops are being installed. The proposed automation scheme is shown in Fig. 8.

This Figure shows the installation location of the control unit. The connection points of the supply loops are shown schematically, this is mainly due to the geographical features of the relief and the availability of power supply points.

## Conclusions

The paper analyzes in detail the existing scheme for automating the technological process of oil production from fields with a high content of paraffin. The technological process of cleaning downhole equipment from Asphalt-resinous and paraffin deposits is considered. The cause-and-effect relationships of deposit formation were also considered. On the basis of the work done, conclusions were drawn about the effectiveness of the use of thermal control methods, but also their high cost. Further, mathematically calculated, and then implemented in the form of technical devices, elements of automated process control systems that provide a more effective technology for combating and preventing the formation of Asphalt-resinous and paraffin deposits. The main tasks implemented in the framework of the study include:

1. A analysis of the scheme of the technological process for the production of high-paraffin oil from fields with a low flow rate was carried out, within the framework of which: research of temperature fields and synthesis of pulsed control of the temperature field based on the Green's function of the wall of a multi-section heater were carried out, taking into account the spatial configuration of the pump-compressor pipes; a mathematical model was built and a system for diagnosing temperature deformation of a tubing due to heat exchange processes was synthesized; developed a method for the optimal location of heating elements on the tubing wall, based on the specified measurement error; a scheme for automating the technological process of extraction of high-paraffin oil from marginal deposits was developed. Also, specialized software has been developed that implements the functioning of the technical part of the presented study. A method has been developed for determining absolute stability based on the Nyquist criterion for impulsive distributed systems for which there is a fundamental solution in the form of a Green's function. Substantiation and development, based on a mathematical model, of recommendations for the smallest number of heating elements integrated into the structure of oilfield tubing.
2. As part of the study, a number of devices were developed that ensure the automation of the technological process, namely: a tubing with impulse sectional heaters was developed; a number of topologies of integrated circuits have been developed to ensure the operation of tubing with pulsed sectional heaters; a number of heating elements with impulse sectional heaters has been developed.
3. Specialized software has been developed for modeling and functioning of the schemes of the developed technological process.

Thus, all the tasks set in this study were completed in full. The resulting technical devices have passed pilot tests.

## Supplementary Information

Supplementary Information 1.

Supplementary Video 1.

## Data Availability

All data generated or analysed during this study are included in this published article and its supplementary information files. Request for more details to the corresponding author.
